# Supplementary material for: Conditional chemoconnectomics (cCCTomics) as a strategy for efficient and conditional targeting of chemical transmission
Source: eLife. 2024 Apr 30;12:RP91927. doi: 10.7554/eLife.91927 (PMC11060718; doi:10.7554/eLife.91927)
Supplement: Figure 3—source data 3. [file elife-91927-fig3-data3.zip › Figure 3 source data3 part 2.zip!/Figure 3-Source data 3-part2/1060f2-1-CRISPResso_Report_gl5umwyq.zip!/CRISPResso_on_1060f2_2/2a.Nucleotide_percentage_quilt.pdf]

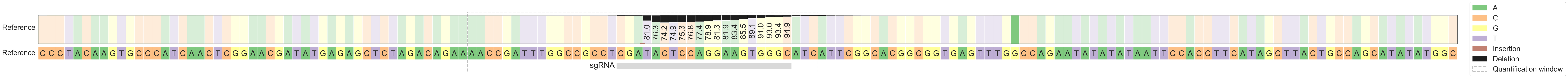
Reference
81.0 76.3 74.2 74.9 75.3 76.8 77.4 78.9 81.3 81.9 83.4 85.5 89.1 91.0 93.0 93.4 94.9
Reference CCCTACAAGTGCCCATCAACTCGGAACGATATGAGAGCTCTAGACAGAAAACCGATTTGGCCGCCTCGATACTCCAGGAAGTGGGCATCATTCGGCACGGCGGTGAGTTTGGCCAGAATATATATAATTCCACCTTCATAGCTTACTGCCAGCATATATGGC
sgRNA
A
C
G
T
Insertion
Deletion
Quantification window
